# MiR-422a as a Potential Cellular MicroRNA Biomarker for Postmenopausal Osteoporosis

**DOI:** 10.1371/journal.pone.0097098

**Published:** 2014-05-12

**Authors:** Zheng Cao, Benjamin T. Moore, Yang Wang, Xian-Hao Peng, Joan M. Lappe, Robert R. Recker, Peng Xiao

**Affiliations:** 1 Department of Orthopedics, The Second Xiangya Hospital, Central South University, Changsha, Hunan, PR China; 2 Osteoporosis Research Center, School of Medicine, Creighton University, Omaha, Nebraska, United States of America; Central China Normal University, China

## Abstract

**Background:**

MicroRNAs (miRNAs) are a class of short non-coding RNA molecules that regulate gene expression by targeting mRNAs. Recently, miRNAs have been shown to play important roles in the etiology of various diseases. However, little is known about their roles in the development of osteoporosis. Circulating monocytes are osteoclast precursors that also produce various factors important for osteoclastogenesis. Previously, we have identified a potential biomarker miR-133a in circulating monocytes for postmenopausal osteoporosis. In this study, we aimed to further identify significant miRNA biomarkers in human circulating monocytes underlying postmenopausal osteoporosis.

**Methodology/Principal Findings:**

We used ABI TaqMan miRNA array followed by qRT-PCR validation in human circulating monocytes from 10 high BMD and 10 low BMD postmenopausal Caucasian women to identify miRNA biomarkers. MiR-422a was up-regulated with marginal significance (P = 0.065) in the low compared with the high BMD group in the array analysis. However, a significant up-regulation of miR-422a was identified in the low BMD group by qRT-PCR analysis (P = 0.029). We also performed bioinformatic target gene analysis and found several potential target genes of miR-422a which are involved in osteoclastogenesis. Further qRT-PCR analyses of the target genes in the same study subjects showed that the expression of five of these genes (CBL, CD226, IGF1, PAG1, and TOB2) correlated negatively with miR-422a expression.

**Conclusions/Significance:**

Our study suggests that miR-422a in human circulating monocytes (osteoclast precursors) is a potential miRNA biomarker underlying postmenopausal osteoporosis.

## Introduction

Osteoporosis is a common disease characterized by low bone mineral density (BMD) and low trauma fractures, mainly resulting from exceeding bone resorption by osteoclasts over bone formation by osteoblasts [1]. The immune system also has a strong association with bone metabolism [2], [3]. In particular, circulating monocytes are directly involved in osteoclastogenesis. They are precursors of osteoclasts [4] and also secrete osteoclastogenic factors such as IL-1 (interleukin-1), IL-6 and TNF-α (tumor necrosis factor-alpha) [5].Human studies have also shown relationships between the expression levels of certain genes in circulating monocytes and osteoporosis: annexin A2 (ANXA2) [6], signal transducer and activator of transcription 1 (STAT1) [7], chemokine (C-C motif) receptor 3 (CCR3), histidine decarboxylase (HDC), and glucocorticoid receptor (GCR) [8].

MicroRNAs (miRNAs) are short (∼22 nt) non-coding RNA molecules that regulate gene expression usually by destabilizing mRNAs or by suppressing translation [9]. Many miRNAs are involved in osteoblastogenesis [10]–[15]. There are also some miRNAs that promote proliferation and differentiation of osteoclasts. MiR-21 downregulates PCD4 (programmed cell death 4) protein expression and induces osteoclastogenesis of primary mouse BMMs (bone marrow-derived monocyte/macrophage precursors) [16]. MiR-155 can induce osteoclast activity via putatively predicted targeting of SHIP (Src homology 2-containing inositol phosphatase), a suppressor of osteoclastogenesis [17]. MiR-223 suppresses NF1-A (nuclear factor 1-A) expression, which stimulates mouse osteoclast differentiation and function [18]. MiR-34c enhances osteoclastogenesis via post-transcriptional regulation of genes involved in the Notch signaling pathway, such as Notch1, Notch2, and Jag1 [19]. MiR-378 is involved in the induction of osteoclast differentiation since it is upregulated in mouse RAW264.7 cells that are differentiating into osteoclasts [20].

However, the role of miRNAs in *in vivo* human circulating monocytes in the etiology of osteoporosis remains largely unclear. A recent *in vitro* study found that miR-146a inhibited osteoclastogenesis in human circulating mononuclear cells [21]. Also, another *in vitro* and *in vivo* study found that miR-148a promotes osteoclastogenesis in human circulating mononuclear cells [22]. We previously found that *in vivo* miR-133a in circulating monocytes is upregulated in postmenopausal women with low BMD compared to postmenopausal women with high BMD, thus identifying miR-133a a potential biomarker associated with postmenopausal osteoporosis [23]. In this study, we intended to further identify other potential miRNA biomarkers in circulating monocytes for postmenopausal osteoporosis. Using microarray and qRT-PCR approaches, we found a significant increase of miR-422a expression in low BMD compared with high BMD subjects. Moreover, bioinformatic target gene analysis identified several possible target genes for miR-422a.

## Materials and Methods

### Human subjects and characteristics

The Institutional Review Board at Creighton University approved the study, and all subjects signed informed-consent documents. Twenty postmenopausal Caucasian women were recruited from the Omaha, NE. Ten subjects had high BMD (spine or hip Z score >0.84) and 10 had low BMD (spine or hip Z score <−0.84). The high and low BMD groups represent the top and bottom 20% of the BMD distributions of the age-, sex- and ethnicity-matched population. BMD (g/cm^2^) for the lumbar spine (L1-4) and total hip (femoral neck, trochanter and intertrochanteric region) were measured using a Hologic 4500A dual energy X-ray absorptiometry (DXA) scanner (Hologic Inc., Bedford, MA). The coefficient of variation (CV), which reflects the instrument's precision, was 0.9% and 1.4% for the spine and hip BMD, respectively. The recruited women were considered postmenopausal if they had at least 12 months of no menses since the date of their last menses. All subjects were 57–68 years of age. Detailed characteristics of the study subjects can be found in our previous publication [23].

### Monocyte isolation

Blood mononuclear cells (MNCs) were isolated from 70 ml peripheral blood from each subject using a density gradient in UNI-SEP tubes containing 5.6% polysucrose and 9.6% sodium metrizoate with a density of 1.077 g/ml (Novamed, Jerusalem, Israel). Monocytes were isolated by negative selection using the untouched human monocytes negative isolation kit (Dynal Biotech, Lake Success, NY), which contains a cocktail of antibodies for CD2, CD7, CD16, CD19, CD56 and CD235a to deplete T cells, granulocytes, B cells, natural killer cells and granulocytes, which leaves the monocytes naïve and untouched to antibodies and microbeads. The purity of isolated monocytes was assessed by flow cytometry using the fluorescence labeled antibodies anti-CD-45-FITC and anti-CD-14-PE [7], [8] (BD Biosciences, San Jose, CA). The average purity is about 85% with 3% deviation.

### Total RNA extraction

The miRNA Isolation Kit (Ambion, Austin, TX) was used to extract total RNA (including miRNA) from each cell sample following the manufacturer's protocol. Total RNA concentration and integrity were measured by the Agilent 2100 Bioanalyzer (Agilent Technologies, Palo Alto, CA). Each RNA sample had quality and an excellent RNA integrity number (>9.0).

### MiRNA array procedures

TaqMan Human MicroRNA Array v1.0 (Applied Biosystems, Foster City, CA) was used to perform a miRNA expression profile for each monocyte RNA sample. Each array tests for the expression of 365 miRNAs and the endogenous controls RNU48 and RNU44. First, TaqMan miRNA Multiplex Reverse Transcription Kit (Applied Biosystems) was used to perform reverse transcription (RT) of the total RNA samples. Each RT reaction was performed in a 63 µl reaction system: 1.8 µl 100 mM dNTPs, 18 µl reverse transcriptase (50 U/ml), 9 µl 10× RT buffer, 1.13 µl RNase inhibitor (20 U/µl), 16 µl RNA sample and 17.08 µl nuclease-free water. The reaction conditions were: 16°C for 30 min, 42°C for 30 min and 85°C for 5 min. Following the RT reaction, 450 µl diluted RT reaction product (diluted 62.5-fold) was mixed with 450 µl TaqMan Universal PCR master mix (Applied Biosystems), and 100 µl of the qRT-PCR reaction mix was loaded into each port of the array card (8 ports/card). The qRT-PCR for each array was performed on the 7900HT Fast Real-Time PCR System (Applied Biosystems). The reaction conditions were: 50°C for 2 min, 95°C for 10 min, and 40 cycles of 95°C for 15 sec followed by 60°C for 1 min. Each array card had only one probe for each target miRNA.

For the miRNA array data analysis, the raw expression level was determined by the cycle number at which the reaction crossed a predetermined threshold cycle (CT) as identified for each miRNA probe. The relative quantity (RQ) of each miRNA for each sample is determined by the calculating 2^−ΔΔCT^, where ΔCT = CT_target_ _miRNA_-CT_endogenous_ _control_ _RNU48_, and ΔΔCT = ΔCT-average ΔCT of all the samples). The RQ values were used for the student's *t* test to identify the miRNAs that were differentially expressed between the high and low BMD groups.

For the array experiments, we did not produce any new data and the array data can be found in our previously published paper [23].

### qRT-PCR for miRNA validation

Due to the possibility of false positive results and to correct for multiple-testing comparison in the miRNA array analysis, we performed qRT-PCR to validate the results obtained in the array. First, RT was performed on the same 20 monocyte RNA samples that were used in the array analysis. The RT reaction was performed with a volume of 15 µl for each sample, using reagents from Applied Biosystems. The contents of each sample included: 1.5 µl TaqMan RT Buffer (10X), 0.15 µl 100 mM dNTPs, 1 µl reverse transcriptase, 0.19 µl RNase inhibitor (20 U/µl), 3 µl miRNA primer, 100 ng total RNA and nuclease-free water to make the final volume 15 µl. The real-time PCR analysis was done on the 7900HT Fast Real-Time PCR System, using 20 µl per sample for the reaction. The contents of each sample were: 2.5 µl cDNA, 10 µl TaqMan universal PCR master mix (2X), 1 µl 20× TaqMan miRNA assay (primers & probe) and 6.5 µl nuclease-free water. The reaction conditions were: 50°C for 2 min, 95°C for 10 min, and 40 cycles of 95°C for 15 sec followed by 60°C for 1 min. For each sample, the target miRNA and RNU48 were run in triplicate on the same plate. The RQ of each miRNA for each sample is determined by the calculating 2^−ΔΔCT^, where ΔCT = CT_target_ _miRNA_-CT_endogenous_ _control_ _RNU48_, and ΔΔCT = ΔCT-average ΔCT of all the samples). The RQ values were used for the student's *t* test to identify the miRNAs that were differentially expressed between the high and low BMD groups.

### Target gene prediction and verification

We performed bioinformatic sequence analysis of each miRNA which showed differential expression between the low and high BMD groups in qRT-PCR validation. Using the miRNA target gene database TargetScan (http://www.targetscan.org), we looked for conserved 8-mer and 7-mer sites in the 3′-untranslated region (UTR) of the target mRNA that are complementary to the seed sequence of the miRNA. This method is used by currently available miRNA target gene databases because miRNAs normally repress gene expression by base pairing at complementarity sites mainly in the 3′-UTR of the target mRNAs [24], [25]. When potential target genes were predicted, we performed qRT-PCR to check for mRNA expression levels of these genes among the same 20 samples. Similar to miRNA qRT-PCR, the mRNA qRT-PCR was also composed of RT and real-time qPCR. The RT and qPCR reaction volumes were 100 µl and 25 µl, respectively, using reagents and following a standard protocol from Applied Biosystems. The RQ calculation was determined by 2^−ΔΔCT^ from the triplicate reaction data of both the target mRNA and internal β-actin control. The student's *t* test was performed to check for potential differential expression levels of the target mRNA between the low and high BMD groups. Furthermore, Pearson correlation analysis was performed between the qRT-PCR expression levels of the miRNA and its potential target genes.

## Results

### MiRNA array analyses

Although the expression levels of the 365 miRNAs were tested in each of the 20 monocyte RNA samples in each individual array, some miRNAs were undetectable in some of the samples. Therefore, we only performed data analyses on the miRNAs which were expressed in at least five high BMD samples and at least five low BMD samples. Using this criterion, statistical analyses were performed for 156 miRNAs. As we previously reported, two miRNAs- miR-133a and miR-382- showed a statistically significant difference in array analysis between the low and high BMD groups [23]. Particularly, miR-133a displayed a fold change of 6.48 between the low and high BMD groups as mean ± SD (4.21±2.15 vs. 0.65±0.75, P = 0.007), and miR-382 showed a fold change of 3.65 between the low and high BMD groups (2.74±2.18 vs. 0.75±0.63, P = 0.027). However, only miR-133a was validated by qRT-PCR (2.21±2.08 vs. 0.76±0.37, P = 0.044), and the qRT-PCR expression levels of miR-382 were not significant (6.56±2.84 vs. 7.93±9.73, P = 0.67). In this study, we further selected four marginal differentially expressed miRNAs in the array study [23] for qRT-PCR validations, which are miR-27b, miR-422a, miR-151, and miR-152. Specifically, the expression levels of the four miRNAs between the low and high BMD groups as mean ± SD in the array analyses are miR-27b (2.48±2.05 vs. 1.02±0.86, P = 0.054), miR-422a (2.91±2.55 vs. 1.24±0.79, P = 0.065), miR-151 (1.44±0.87 vs. 0.86±0.42, P = 0.076), and miR-152 (1.32±0.49 vs. 0.91±0.47, P = 0.076) (Figure S1).

### MiRNA qRT-PCR

**Figure 1:**
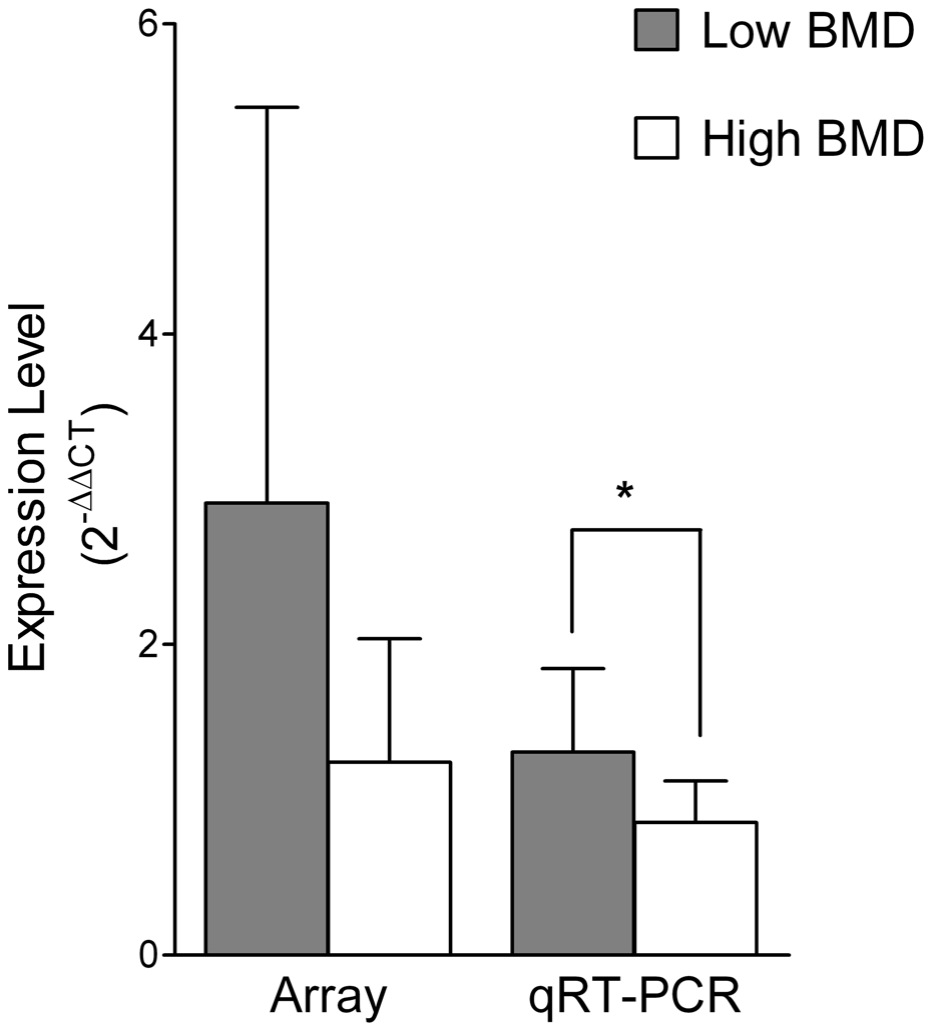
MiR-442a expression levels in human circulating monocytes from postmenopausal women with low and high BMD as shown by miRNA array and qRT-PCR. (*P<0.05, N = 20)

To further validate the differential expression levels of the four selected miRNAs between the low and high BMD groups, we performed qRT-PCR for the four miRNAs on the 20 monocyte RNA samples. Only miR-422a showed a significant upregulation in the low BMD group as compared to the high BMD group (1.31±0.54 vs. 0.85±0.27, P = 0.029) was confirmed by qRT-PCR (Figure 1). The expression levels in the low vs. the high BMD group of miR-27b (1.26±0.66 vs. 0.99±0.43, P = 0.29), miR-151 (1.37±1.08 vs. 0.98±0.41, P = 0.30) and miR-152 (1.50±1.11 vs. 0.95±0.54, P = 0.17) were not significant (Figure S2).

### Target gene prediction and verification

**Figure 2:**
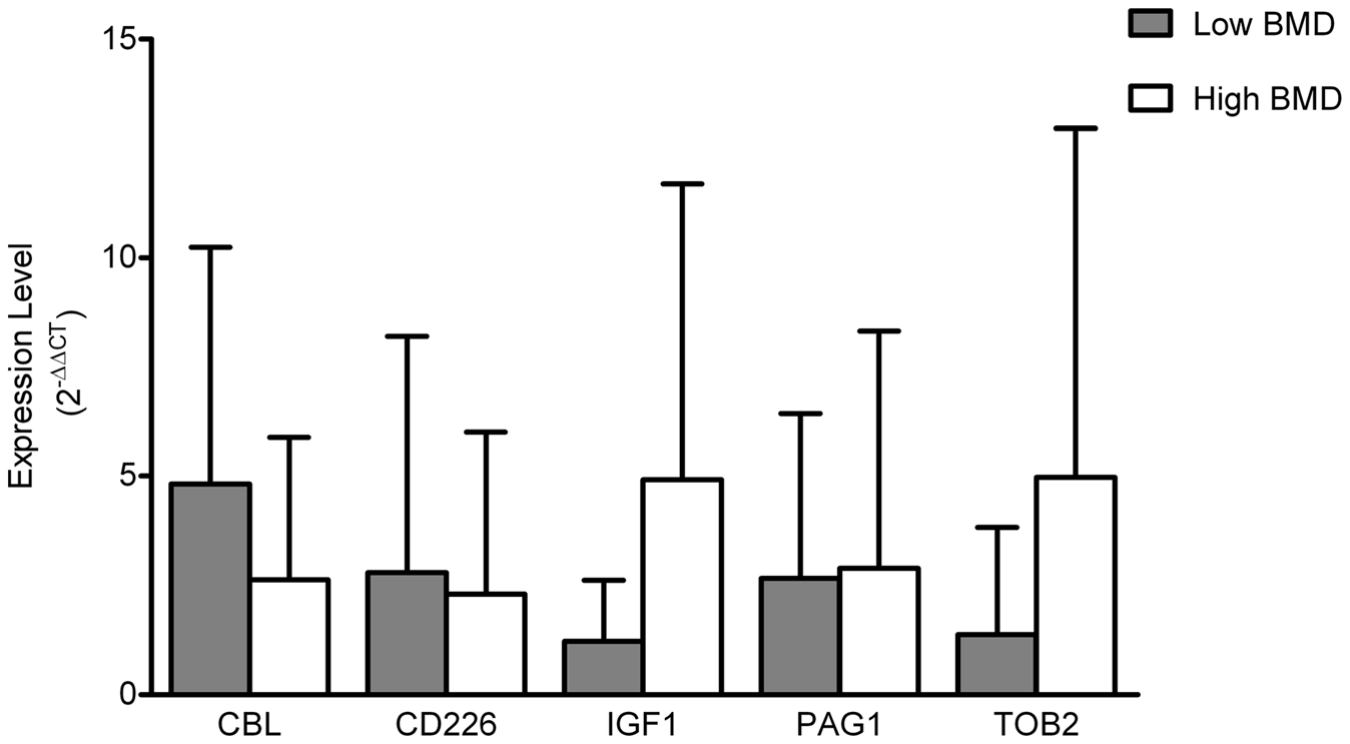
Expression levels of potential miR-422a target genes in human circulating monocytes from postmenopausal women with low and high BMD as shown by qRT-PCR. (P>0.05, N = 20)

Our search for predicted target genes of miR-422a found 191 potential targets using TargetScan database. We found several genes that are potential miR-422a targets and are related to osteoclastogenesis: CBL (Casitas B-lineage lymphoma proto-oncogene)http://www.ncbi.nlm.nih.gov/entrez/query.fcgi?db=gene&cmd=Retrieve&dopt=full_report&list_uids=867, CD226 (cluster of differentiation 226), IGF1 (insulin-like growth factor 1), PAG1 (phosphoprotein associated with glycosphingolipid microdomains 1) and TOB2 (transducer of ERBB2, 2). The specific binding sites of miR-422a to the 3′ UTR regions of these five genes are shown in Table 1. qRT-PCR analyses of these five genes did not demonstrate significant difference in mRNA expression between the low and high BMD groups (Figure 2). Correlation analysis between miR-422a and these five potential target genes showed a negative correlation between each of the five genes and miR-422a, although none of the correlations were significant (P>0.05) (Table 2).

**Table 1:** Putative binding sites of miR-422a in predicted target genes in humans.

Target Gene	3' UTR Position	Consequential Pairing Target gene binding region (top) and miR-422a sequence (bottom)
CBL		5'CCUACCUGGGGAAC**AGUCCAG**A
	283–290	
		3'CGGAAGACUGGGAU**UCAGGUC**A
CD226		5'CACUCUAUCAGCAU**AGUCCAG**A
	776–783	
		3'CGGAAGACUGGGAU**UCAGGUC**A
IGF1		5'CAAAUUG**GAC**UGGCG**AGUCCAG**A
	1114–1121	
		3'CGGAAGA**CUG**GGAU-**UCAGGUC**A
	4802-4809	5'UUGCAAA**GACC**AUAA**AGUCCAG**A
		
		3'CGGAAGA**CUGG**GAU-**UCAGGUC**A
PAG1		5'AACUUAAAGAGAUC**AGUCCAG**A
	4605–4612	
		3'CGGAAGACUGGGAU**UCAGGUC**A
TOB2		5'GAAUGGAAACUGUU**AGUCCAG**A
	2506–2513	
		3'CGGAAGACUGGGAU**UCAGGUC**A

**Table 2:** Pearson correlation of expression levels (2^−ΔΔCT^) of miR-422a with potential target genes as measured by qRT-PCR.

Gene	Fold Change (H/L)	Correlation Coefficient	P Value
CBL	0.544143	−0.18871	0.4256
CD226	0.822103	−0.15951	0.5017
IGF1	4.016441	−0.06055	0.8055
PAG1	1.08477	−0.12803	0.6119
TOB2	3.60556	−0.04008	0.8668

Note: H/L: high/low BMD groups.

## Discussion

The purpose of this study was to further identify miRNA biomarkers in human circulating monocytes associated with postmenopausal osteoporosis. Our previous work showed a significant upregulation of miR-133a in circulating monocytes in the low vs. the high BMD postmenopausal women by both array and qRT-PCR analyses and identified miR-133a as a potential biomarker for postmenopausal osteoporosis. In this study, to avoid potential false negative results, we performed further data mining of the array data and selected four marginal significant miRNAs for qRT-PCR validation and target gene analyses. We found another significant miR-422a in circulating monocytes as a potential cellular miRNA biomarker underlying postmenopausal osteoporosis.

Human mature miR-422a is encoded by gene MIR422A at 15q22.31 (64,163,129–64,163,218 bp). The array results demonstrated that miR-422a was marginally unregulated in the low vs. the high BMD groups (P = 0.065). However, further qRT-PCR validation found a significant upregulation of miR-422a in the low vs. the high BMD group (P = 0.029) (Figure 1). In addition, we detected miR-422a expression levels in circulating B cells from the same 20 high or low BMD postmenopausal women. Circulating B cells were isolated by Dynabeads CD19 (Pan B) (Dynal Biotech). However, miR-422a was not differentially expressed in B cells between the high and the low BMD groups (P = 0.31). Therefore, miR-422a is another potential monocyte-specific biomarker for postmenopausal osteoporosis.

There have been some studies to determine the importance of miR-422a in human diseases. MiR-422a may play a protective role against colon cancer, which was shown by its decreased expression in colorectal tumor or in laryngeal carcinoma tissue as compared to normal tissues [26], [27]. MiR-422a also inhibits pathways that stimulate tumor cell proliferation in osteosarcoma [28]. MiR-422a may have a deleterious effect in individuals with multiple sclerosis (MS), and plasma from individuals with MS showed significantly higher levels of miR-422a than plasma from healthy subjects [29]. MiR-422a also destabilizes CYP7A1 mRNA by binding to the 3′ UTR and affects CYP7A1 gene's role in bile acid synthesis [30]. MiR-422a was co-expressed in human monocytes and atherosclerotic plaque tissue [31]. In bone research area, only one study has shown that the treatment of osteoblasts with peptide-15, which is known to increase bone formation, decreased expression of miR-422a in osteoblasts-like cells [32]. Our current study, however, has initially demonstrated that miR-422a in human circulating monocytes, the osteoclast precursors, is associated with potemenopausal BMD levels.

This study identified miR-422a as a potential biomarker for postmenopausal osteoporosis. However, the specific mechanism of miR-422a in circulating monocytes to reduce BMD is still unknown. Since circulating monocytes are osteoclast precursors, we have tried to further identify potential miR-422a target genes that inhibit osteoclastogenesis. By searching miRNA target gene predicting databases (TargetScan) and available published references, we found four potential miR-422a target genes related to the inhibition of osteoclastogenesis, which are CBL, CD226, PAG1, and TOB2 (Table 1). CBL inhibits early stage osteoclastogenesis by degrading NFATc1 (nuclear factor of activated T cells c1) protein [33], CD226, also called DNAM-1 (DNAX accessory molecule-1), has been found to negatively regulate osteoclast formation [34], PAG1 has an alias of CBP (Csk-binding protein). An *in vitro* study has demonstrated that up-regulation of CBP dramatically inhibited bone-resorbing activity in osteoclasts http://www.genenames.org/data/hgnc_data.php?hgnc_id=30043 [35]. TOB2 inhibits formation of osteoclasts by interacting with VDR (vitamin D receptor) to suppress RANKL expression [36]. In our study, miR-422a has been up-regulated in the postmenopausal low BMD group. Therefore, the increased miR-422a in circulating monocytes may inhibit the four inhibitory factors for osteoclastogenesis, and thus stimulate osteoclastogenesis and result in low BMD by more bone resportion in postmenopausal women. Another important gene that may be inhibited by miR-422a is IGF1. Many studies have found that circulating levels of IGF1 are positively associated with BMD in both men and women, and osteoporotic patients have reduced circulating IGF1 levels [37]–[39]. A recent review by Crane and Cao has also indicated that IGF1 is a critical growth factor to maintain bone mass by its role in the coupled bone remodeling [40]. In this study, the up-regulation of miR-422a may reduce the production and secretion of IGF1 in circulating monocytes, leading to low BMD and osteoporosis in postmenopausal women. Therefore, all the five potential target genes play beneficial roles in bone metabolism and down-regulation of the five genes should be related to low BMD and osteoporosis. In this study, the qRT-PCR analyses in the same 20 circulating monocyte RNA samples have not shown significant differential expression levels of the five potential target genes between the high and low BMD group (Figure 2). However, miR-422a expression levels have negative correlations with all the five genes though not significant (Table 2). It is possible that miR-422a inhibits each of the five target genes in small extension but none of the gene expressions individually are significantly different between the low and high BMD groups. However, the combined systematic effects may be significant and result in bone loss and low BMD. Alternatively, one of the main miRNA regulatory mechanisms is to suppress protein translation only but not to induce mRNA degradation [24], [25], which maybe another reason for the non-significant differentiation and correlation of the target genes at the mRNA level. Moreover, since there are 226 predicted target genes of miR-422a in TargetScan database, there may be some novel bone-related genes that are still unknown. In addition, our sample size (N = 20) is relatively small. A larger sample size will have higher statistical power to identify potential target genes with small effect sizes. To find out if there is any relationship between the two significant miRNA markers that we identified in our current and previous studies, we also performed Pearson correlation analysis between the expression levels of miR-422a and miR-133a. The results showed a positive correlation but not statistically significant (Pearson Correlation Coefficient  = 0.1685, P = 0.4777). As our research continues, we will also use standard techniques, such as logistic regression and/or CARTmodel, to combine a series of interested miRNAs for biomarker-based classification in our future studies.

Therefore, following our initial miRNA biomarker study in human circulating monocytes underlying postmenopausal osteoporosis, this study further identified miR-422a as a specific cellular marker for postmenopausal osteoporosis.

## Supporting Information

Figure S1 Expression levels of miR-27b, miR-151 and miR-152 in human circulating monocytes from postmenopausal women with low and high BMD as shown by miRNA array. (P>0.05, N = 20).
(TIF)

Figure S2 Expression levels of miR-27b, miR-151 and miR-152 in human circulating monocytes from postmenopausal women with low and high BMD as shown by qRT-PCR. (P>0.05, N = 20).
(TIF)

## References

[1] Manolagas SC (2000) Birth and death of bone cells: basic regulatory mechanisms and implications for the pathogenesis and treatment of osteoporosis. Endocr Rev 21: 115–137. 10782361 10.1210/edrv.21.2.0395

[2] Arron JR, Choi Y (2000) Bone versus immune system. Nature 408: 535–536. 11117729 10.1038/35046196

[3] Walsh MC, Kim N, Kadono Y, Rho J, Lee SY, et al (2006) Osteoimmunology: interplay between the immune system and bone metabolism. Annu Rev Immunol 24: 33–63. 16551243 10.1146/annurev.immunol.24.021605.090646

[4] Fujikawa Y, Quinn JM, Sabokbar A, McGee JO, Athanasou NA (1996) The human osteoclast precursor circulates in the monocyte fraction. Endocrinology 137: 4058–4060. 8756585 10.1210/endo.137.9.8756585

[5] Cohen-Solal ME, Graulet AM, Denne MA, Gueris J, Baylink D, et al (1993) Peripheral monocyte culture supernatants of menopausal women can induce bone resorption: involvement of cytokines. J Clin Endocrinol Metab 77: 1648–1653. 8263153 10.1210/jcem.77.6.8263153

[6] Deng FY, Lei SF, Zhang Y, Zhang YL, Zheng YP, et al (2011) Peripheral blood monocyte-expressed ANXA2 gene is involved in pathogenesis of osteoporosis in humans. Mol Cell Proteomics 10: M111. 10.1074/mcp.M111.011700 PMC3226411 21817168

[7] Chen XD, Xiao P, Lei SF, Liu YZ, Guo YF, et al (2010) Gene expression profiling in monocytes and SNP association suggest the importance of the STAT1 gene for osteoporosis in both Chinese and Caucasians. J Bone Miner Res 25: 339–355. 19594299 10.1359/jbmr.090724 PMC3153389

[8] Liu YZ, Dvornyk V, Lu Y, Shen H, Lappe JM, et al (2005) A novel pathophysiological mechanism for osteoporosis suggested by an in vivo gene expression study of circulating monocytes. J Biol Chem 280: 29011–29016. 15965235 10.1074/jbc.M501164200

[9] Ebert MS, Sharp PA (2012) Roles for microRNAs in conferring robustness to biological processes. Cell 149: 515–524. 22541426 10.1016/j.cell.2012.04.005 PMC3351105

[10] Li Z, Hassan MQ, Volinia S, van Wijnen AJ, Stein JL, et al (2008) A microRNA signature for a BMP2-induced osteoblast lineage commitment program. Proc Natl Acad Sci U S A 105: 13906–13911. 18784367 10.1073/pnas.0804438105 PMC2544552

[11] Hassan MQ, Gordon JA, Beloti MM, Croce CM, van Wijnen AJ, et al (2010) A network connecting Runx2, SATB2, and the miR-23a∼27a∼24–2 cluster regulates the osteoblast differentiation program. Proc Natl Acad Sci U S A 107: 19879–19884. 20980664 10.1073/pnas.1007698107 PMC2993380

[12] Kapinas K, Kessler C, Ricks T, Gronowicz G, Delany AM (2010) miR-29 modulates Wnt signaling in human osteoblasts through a positive feedback loop. J Biol Chem 285: 25221–25231. 20551325 10.1074/jbc.M110.116137 PMC2919085

[13] Mizuno Y, Yagi K, Tokuzawa Y, Kanesaki-Yatsuka Y, Suda T, et al (2008) miR-125b inhibits osteoblastic differentiation by down-regulation of cell proliferation. Biochem Biophys Res Commun 368: 267–272. 18230348 10.1016/j.bbrc.2008.01.073

[14] Inose H, Ochi H, Kimura A, Fujita K, Xu R, et al (2009) A microRNA regulatory mechanism of osteoblast differentiation. Proc Natl Acad Sci U S A 106: 20794–20799. 19933329 10.1073/pnas.0909311106 PMC2791638

[15] Li H, Xie H, Liu W, Hu R, Huang B, et al (2009) A novel microRNA targeting HDAC5 regulates osteoblast differentiation in mice and contributes to primary osteoporosis in humans. J Clin Invest 119: 3666–3677. 19920351 10.1172/JCI39832 PMC2786801

[16] Sugatani T, Vacher J, Hruska KA (2011) A microRNA expression signature of osteoclastogenesis. Blood 117: 3648–3657. 21273303 10.1182/blood-2010-10-311415 PMC3072882

[17] Mizoguchi F, Izu Y, Hayata T, Hemmi H, Nakashima K, et al (2010) Osteoclast-specific Dicer gene deficiency suppresses osteoclastic bone resorption. J Cell Biochem 109: 866–875. 20039311 10.1002/jcb.22228

[18] Sugatani T, Hruska KA (2009) Impaired micro-RNA pathways diminish osteoclast differentiation and function. J Biol Chem 284: 4667–4678. 19059913 10.1074/jbc.M805777200 PMC2640963

[19] Bae Y, Yang T, Zeng HC, Campeau PM, Chen Y, et al (2012) miRNA-34c regulates Notch signaling during bone development. Hum Mol Genet 21: 2991–3000. 22498974 10.1093/hmg/dds129 PMC3373245

[20] Kagiya T, Nakamura S (2012) Expression profiling of microRNAs in RAW264.7 cells treated with a combination of tumor necrosis factor alpha and RANKL during osteoclast differentiation. J Periodontal Res 48: 373–85. 23078176 10.1111/jre.12017

[21] Nakasa T, Shibuya H, Nagata Y, Niimoto T, Ochi M (2011) The inhibitory effect of microRNA-146a expression on bone destruction in collagen-induced arthritis. Arthritis Rheum 63: 1582–1590. 21425254 10.1002/art.30321

[22] Cheng P, Chen C, He HB, Hu R, Zhou HD, et al (2013) miR-148a regulates osteoclastogenesis by targeting V-maf musculoaponeurotic fibrosarcoma oncogene homolog B. J Bone Miner Res 28: 1180–1190. 23225151 10.1002/jbmr.1845

[23] Wang Y, Li L, Moore BT, Peng XH, Fang X, et al (2012) MiR-133a in human circulating monocytes: a potential biomarker associated with postmenopausal osteoporosis. PLoS One 7: e34641. 22506038 10.1371/journal.pone.0034641 PMC3323546

[24] He L, Hannon GJ (2004) MicroRNAs: small RNAs with a big role in gene regulation. Nat Rev Genet 5: 522–531. 15211354 10.1038/nrg1379

[25] Bartel DP (2004) MicroRNAs: genomics, biogenesis, mechanism, and function. Cell 116: 281–297. 14744438 10.1016/s0092-8674(04)00045-5

[26] Faltejskova P, Svoboda M, Srutova K, Mlcochova J, Besse A, et al (2012) Identification and functional screening of microRNAs highly deregulated in colorectal cancer. J Cell Mol Med 16: 2655–2666. 22469014 10.1111/j.1582-4934.2012.01579.x PMC4118234

[27] Wang P, Fu T, Wang X, Zhu W (2010) Primary, study of miRNA expression patterns in laryngeal carcinoma by microarray. Lin Chung Er Bi Yan Hou Tou Jing Wai Ke Za Zhi 24: 535–538. 20806854

[28] Gougelet A, Pissaloux D, Besse A, Perez J, Duc A, et al (2011) Micro-RNA profiles in osteosarcoma as a predictive tool for ifosfamide response. Int J Cancer 129: 680–690. 20949564 10.1002/ijc.25715

[29] Siegel SR, Mackenzie J, Chaplin G, Jablonski NG, Griffiths L (2012) Circulating microRNAs involved in multiple sclerosis. Mol Biol Rep 39: 6219–6225. 22231906 10.1007/s11033-011-1441-7

[30] Song KH, Li T, Owsley E, Chiang JY (2010) A putative role of micro RNA in regulation of cholesterol 7alpha-hydroxylase expression in human hepatocytes. J Lipid Res 51: 2223–2233. 20351063 10.1194/jlr.M004531 PMC2903801

[31] Bidzhekov K, Gan L, Denecke B, Rostalsky A, Hristov M, et al (2012) microRNA expression signatures and parallels between monocyte subsets and atherosclerotic plaque in humans. Thromb Haemost 107: 619–625. 22370758 10.1160/TH11-09-0607

[32] Palmieri A, Pezzetti F, Brunelli G, Martinelli M, Lo ML, et al (2008) Peptide-15 changes miRNA expression in osteoblast-like cells. Implant Dent 17: 100–108. 18332763 10.1097/ID.0b013e318166d182

[33] Kim JH, Kim K, Jin HM, Song I, Youn BU, et al (2010) Negative feedback control of osteoclast formation through ubiquitin-mediated down-regulation of NFATc1. J Biol Chem 285: 5224–5231. 20037154 10.1074/jbc.M109.042812 PMC2820750

[34] Kakehi S, Nakahama K, Morita I (2007) Expression and possible role of PVR/CD155/Necl-5 in osteoclastogenesis. Mol Cell Biochem 301: 209–217. 17286202 10.1007/s11010-007-9413-x

[35] Matsubara T, Ikeda F, Hata K, Nakanishi M, Okada M, et al (2010) Cbp recruitment of Csk into lipid rafts is critical to c-Src kinase activity and bone resorption in osteoclasts. J Bone Miner Res 25: 1068–1076. 19874196 10.1359/jbmr.091039

[36] Ajima R, Akiyama T, Usui M, Yoneda M, Yoshida Y, et al (2008) Osteoporotic bone formation in mice lacking tob2; involvement of Tob2 in RANK ligand expression and osteoclasts differentiation. FEBS Lett 582: 1313–1318. 18358842 10.1016/j.febslet.2008.03.012

[37] Gillberg P, Olofsson H, Mallmin H, Blum WF, Ljunghall S, et al (2002) Bone mineral density in femoral neck is positively correlated to circulating insulin-like growth factor (IGF)-I and IGF-binding protein (IGFBP)-3 in Swedish men. Calcif Tissue Int 70: 22–29. 11907704 10.1007/s002230020048

[38] Langlois JA, Rosen CJ, Visser M, Hannan MT, Harris T, et al (1998) Association between insulin-like growth factor I and bone mineral density in older women and men: the Framingham Heart Study. J Clin Endocrinol Metab 83: 4257–4262. 9851760 10.1210/jcem.83.12.5308

[39] Yamaguchi T, Kanatani M, Yamauchi M, Kaji H, Sugishita T, et al (2006) Serum levels of insulin-like growth factor (IGF); IGF-binding proteins-3, -4, and -5; and their relationships to bone mineral density and the risk of vertebral fractures in postmenopausal women. Calcif Tissue Int 78: 18–24. 16397738 10.1007/s00223-005-0163-z PMC2904611

[40] Crane JL, Cao X (2013) Function of matrix IGF-1 in coupling bone resorption and formation. J Mol Med 92: 107–115. 24068256 10.1007/s00109-013-1084-3 PMC3946714

