# Supplementary figures and images for: MiR-422a as a Potential Cellular MicroRNA Biomarker for Postmenopausal Osteoporosis
Source: PLoS One. 2014 May 12;9(5):e97098. doi: 10.1371/journal.pone.0097098 (PMC4018259; doi:10.1371/journal.pone.0097098)

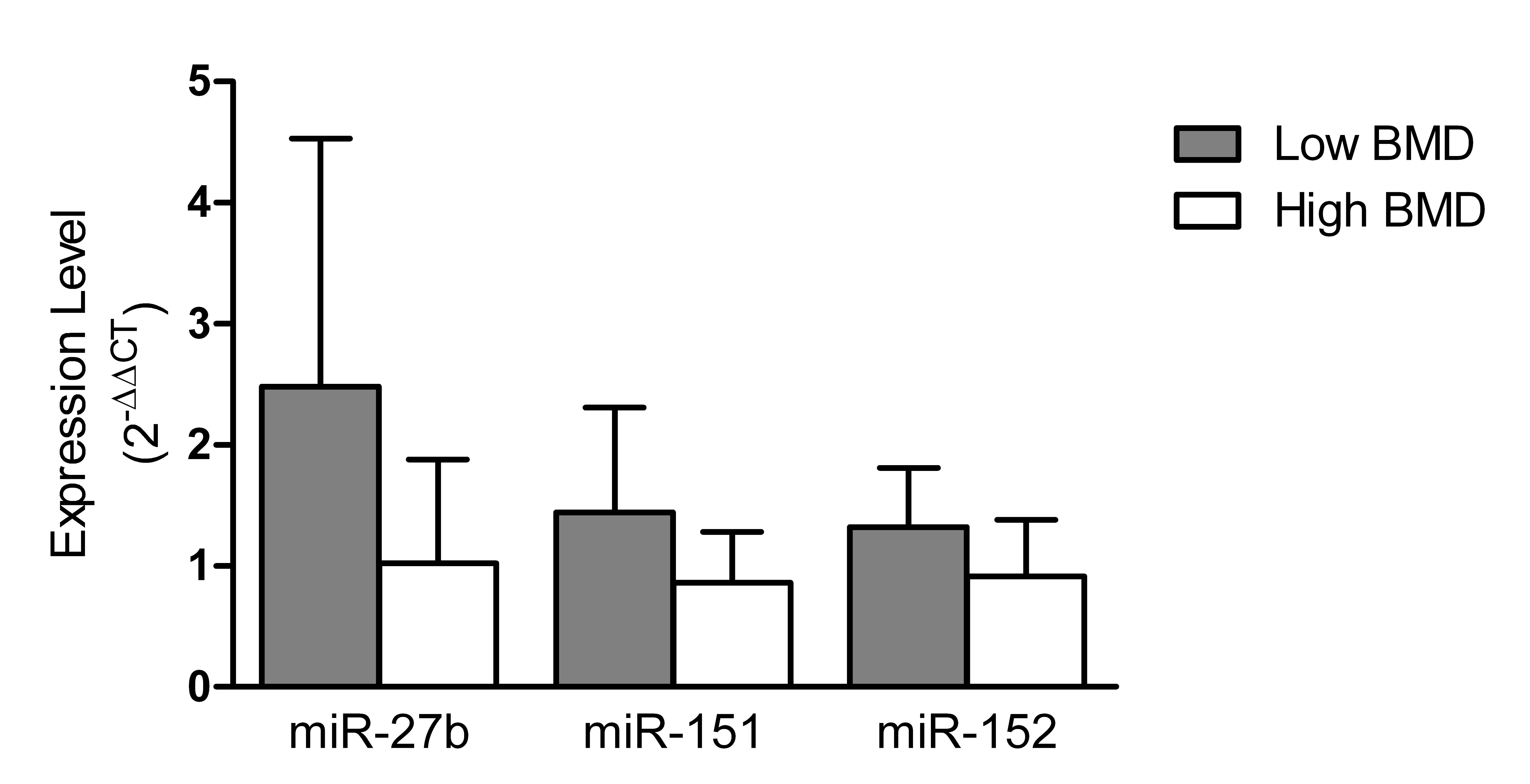

Supplement: Figure S1 — Expression levels of miR-27b, miR-151 and miR-152 in human circulating monocytes from postmenopausal women with low and high BMD as shown by miRNA array. (P>0.05, N = 20). (TIF) [file pone.0097098.s001.tif]

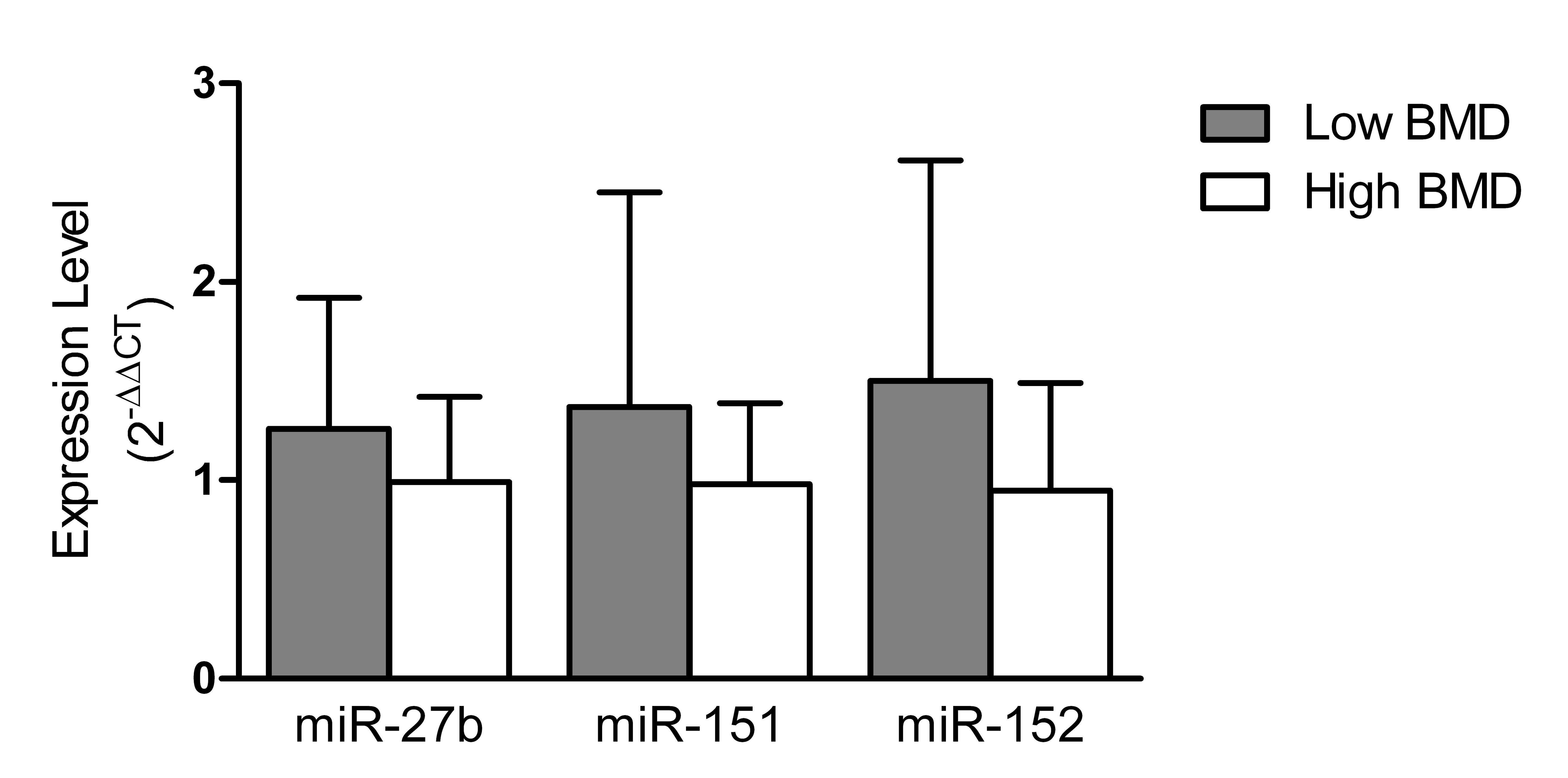

Supplement: Figure S2 — Expression levels of miR-27b, miR-151 and miR-152 in human circulating monocytes from postmenopausal women with low and high BMD as shown by qRT-PCR. (P>0.05, N = 20). (TIF) [file pone.0097098.s002.tif]
